# Supplementary material for: Apelin signaling acts as a molecular switch between endothelial and hematopoietic stem cell fates
Source: EMBO Rep. 2025 Dec 16;27(4):899–924. doi: 10.1038/s44319-025-00656-6 (PMC12936188; doi:10.1038/s44319-025-00656-6)
Supplement: Supplementary file 2 — Expanded View Figures [file 44319_2025_656_MOESM2_ESM.pdf]

Expanded View Figures

Figure EV1. *aplnrb* negative cells are restricted to the VDA.

(**A–A″**) Confocal projection images of the trunk region of *Tg^{BAC}(aplnrb:Venus-PEST); Tg(kdrl:NLS-mCherry)* double transgenic zebrafish embryos at 22 hpf. *aplnrb:*Venus-PEST negative ECs can be detected in the ventral DA (cyan arrowheads). (**B**, **B″**) Surface rendering of a confocal projection image of a double transgenic *Tg^{BAC}(aplnrb:Venus-PEST); Tg(kdrl:NLS-mCherry* embryos at 48 hpf highlighting the absence of *aplnrb:*Venus-PEST expression (cyan) from putative HECs (yellow arrowheads) in the ventral DA (nuclei in magenta). (**C**) Confocal projection image of a double transgenic *Tg^{BAC}(aplnrb:Venus-PEST); Tg(kdrl:NLS-mCherry* larva at 72 hpf. Expression of *aplnrb*:Venus-PEST is absent from the DA and PCV and is restricted to the intersegmental vessels. (**D**, **D′**) Confocal projection images of a *Tg^{BAC}(aplnrb:Venus-PEST); Tg(kdrl:HsHRAS-mCherry* double transgenic zebrafish embryo at 48 hpf. *aplnrb:*Venus-PEST negative ECs can be detected in the ventral DA (cyan arrowheads). (**E**, **E′**) Sagittal slice of the images in (**D**, **D′**). (**F**, **F′**) Confocal projection images of the trunk region of a *Tg(kdrl:GFP); Tg(kdrl:NLS-mCherry)* double transgenic embryo at 48 hpf. All ECs in the DA are marked with *kdrl*:GFP and *kdrl*:NLS-mCherry expression. (**G**, **G′**) Sagittal slice of the images in (**F**, **F′**). (**H**) Quantitative RT-PCR results for *kdrl* on FACS sorted *aplnrb*:Venus-PEST expressing and non-expressing ECs, referring to Fig. 1E–G ($n = 3$, paired t test, $P < 0.0003$). Quantifications are displayed as mean ± SD. (**I–J′**) Confocal projection images of double transgenic *Tg(kdrl:HsHRAS-mCherry); Tg^{BAC}(apln:GFP)* zebrafish embryo at 24 hpf (**I**, **I′**) and embryo at 48 hpf (**J**, **J′**). At both timepoints displayed, no expression of the reporter for *apln* can be detected in the DA and the PCV. The notochord shows *apln* expression that is decreasing over time. DA dorsal aorta, PCV posterior cardinal vein, HECs hemogenic endothelia cells, ECs endothelial cells, NC notochord. Scale bars: 20 µm.

© The Author(s)

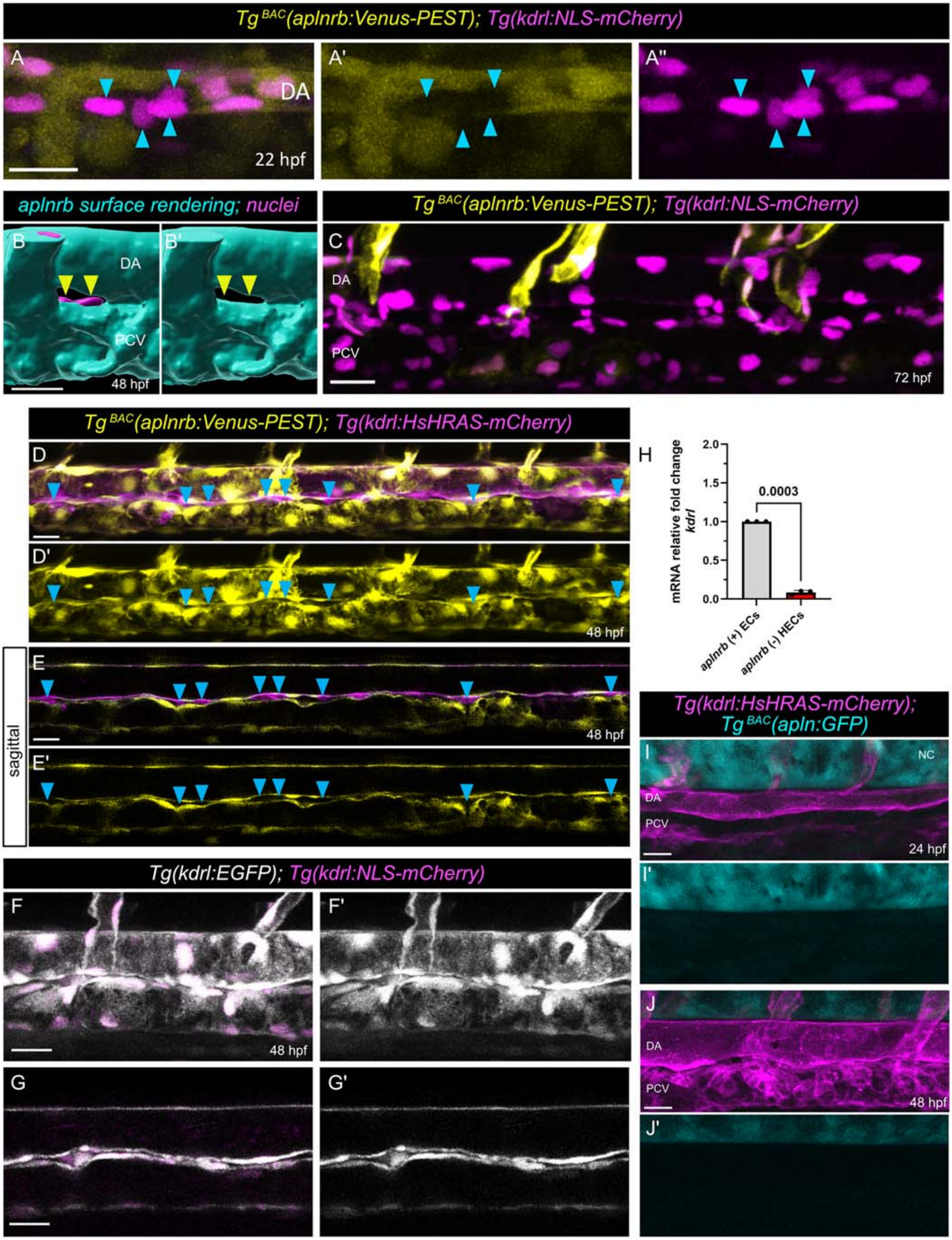
Tg BAC(aplnrb:Venus-PEST); Tg(kdrl:NLS-mCherry)
A
A'
A''
DA
22 hpf
aplnrb surface rendering; nuclei
B
B'
DA
PCV
48 hpf
Tg BAC(aplnrb:Venus-PEST); Tg(kdrl:NLS-mCherry)
C
DA
PCV
72 hpf
Tg BAC(aplnrb:Venus-PEST); Tg(kdrl:HsHRAS-mCherry)
D
D'
48 hpf
E
E'
sagittal
H
mRNA relative fold change kdrl
2.0
1.5
1.0
0.5
0.0
0.0003
aplnrb (+) ECs
aplnrb (-) HECs
Tg(kdrl:HsHRAS-mCherry); Tg BAC(apln:GFP)
I
I'
NC
DA
PCV
24 hpf
Tg(kdrl:EGFP); Tg(kdrl:NLS-mCherry)
F
F'
48 hpf
G
G'
J
J'
DA
PCV
48 hpf

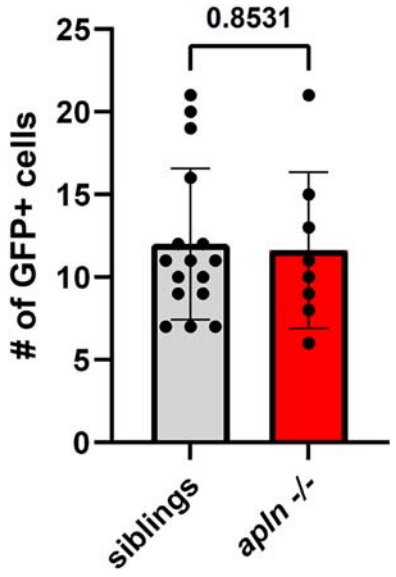

Figure EV2. No detectable change in HSPC numbers in zygotic *apln* mutant embryos.

Quantification of itga2b:GFP expressing cells in the DA at 52 hpf ($n = 32$, unpaired t test, $P = 0.0044$); Quantifications are displayed as mean ± SD.

 © The Author(s)

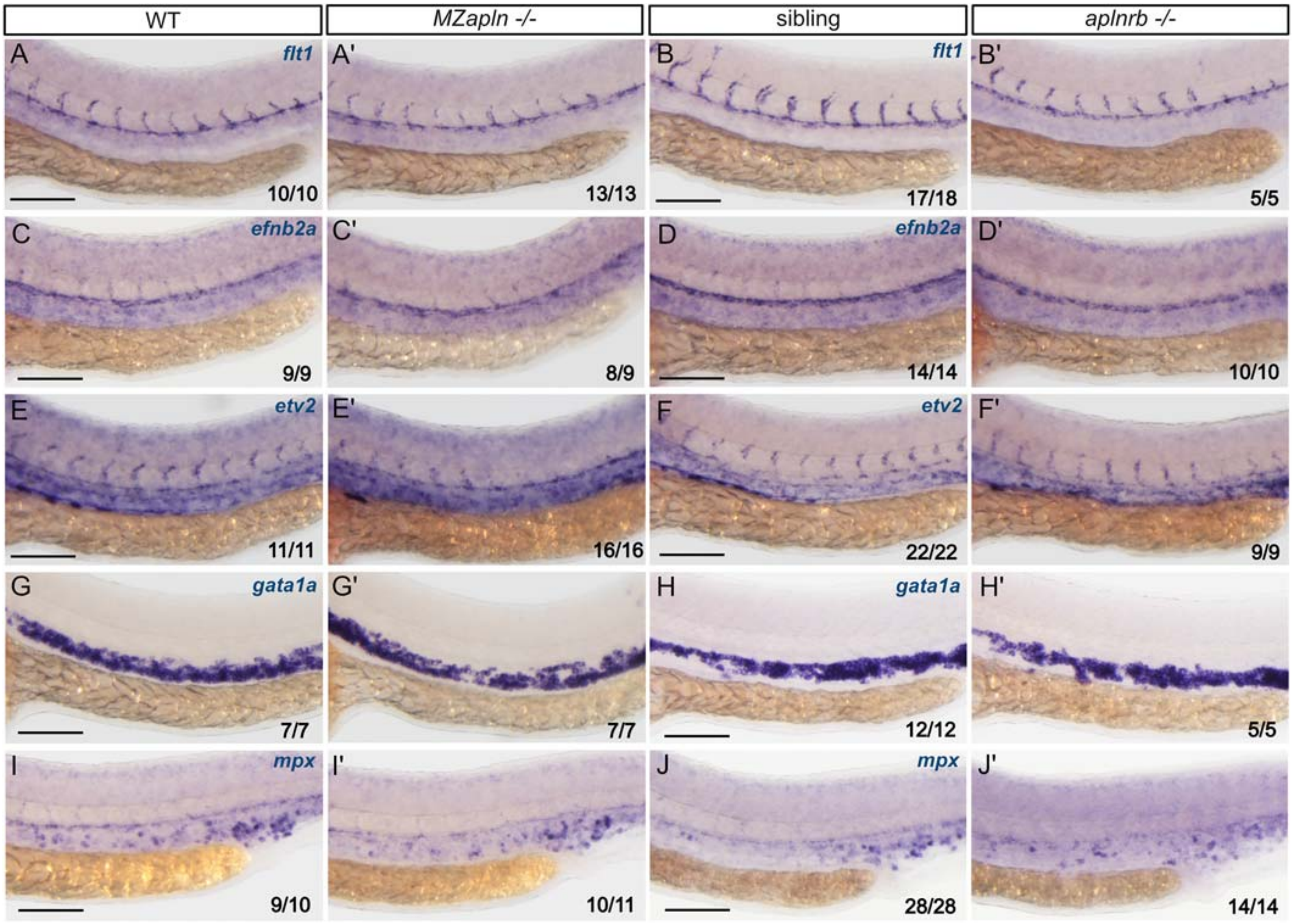

Figure EV3. Arterial differentiation and primitive hematopoiesis are unaltered upon loss of Apelin signaling.

(**A–J′**) WISH on wild-type and *MZapln -/-* mutant (**A**, **A′**, **C**, **C′**, **E**, **E′**, **G**, **G′**, **I**, **I′**) and siblings and *aplnrb -/-* mutant (**B**, **B′**, **D**, **D′**, **F**, **F′**, **H**, **H′**, **J**, **J′**) zebrafish embryos at 24 hpf. (**A–B′**) WISH for *flt1* showing WT-like expression upon loss of Apelin signaling. (**C–D′**) WISH for *efnb2a* showing WT-like expression upon loss of Apelin signaling. (**E–F′**) WISH for *etv2* showing WT-like expression upon loss of Apelin signaling. (**G–H′**) WISH for *gata1a* showing WT-like expression upon loss of Apelin signaling. (**I–J′**) WISH for *mpx* showing WT-like expression upon loss of Apelin signaling. Scale bars: 100 μm.

© The Author(s)

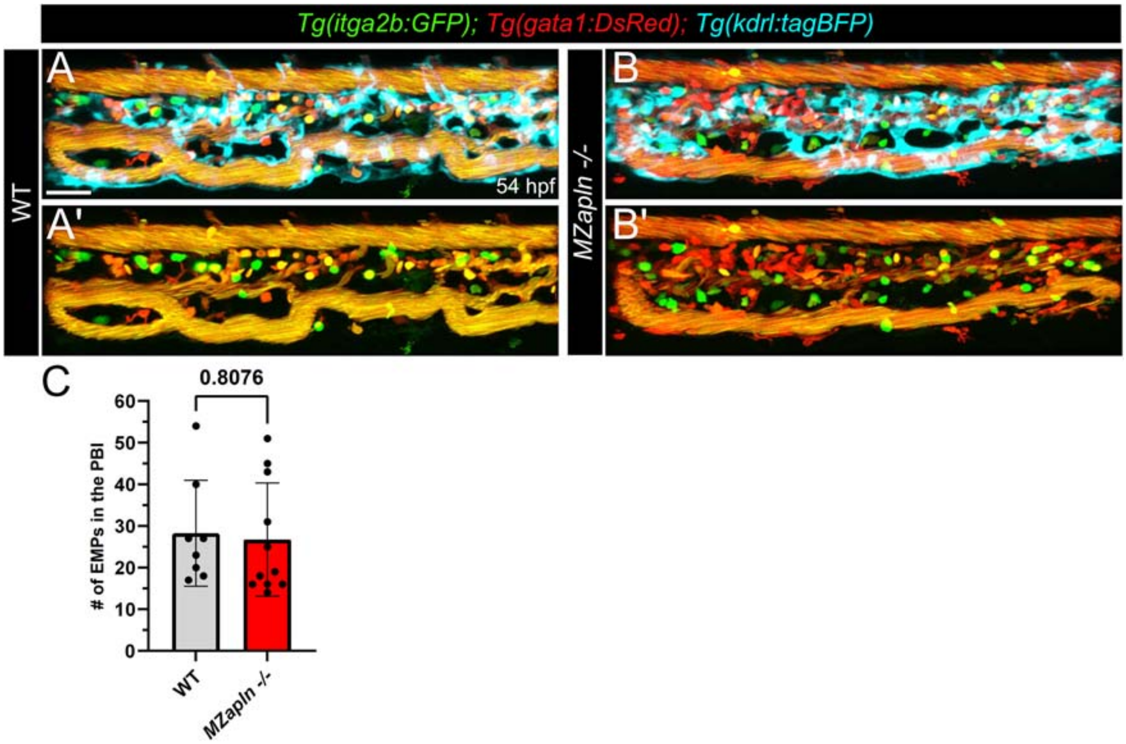

Figure EV4. EMP development is independent of Apelin signaling.

(**A–B'**) Confocal projection images of the CHT region of *Tg(itga2b:GFP); Tg(gata1:DsRed); Tg(kdrl:tagBFP)* triple transgenic zebrafish larvae at 54 and 72 hpf. (**A–B'**) Exemplary images of wild-type and *MZapln* −/− mutant zebrafish larvae at 54 hpf. (**C**) Quantification corresponding to (**A–B'**). *itga2b*:GFP + /*gata1a*:DsRed+ EMPs were counted in the CHT ($n = 19$, unpaired t test, $P = 0.8076$). EMP erythro-myeloid progenitors, CHT caudal hematopoietic tissue. Quantifications are displayed as mean ± SD. Scale bars: 40 μm.

 © The Author(s)

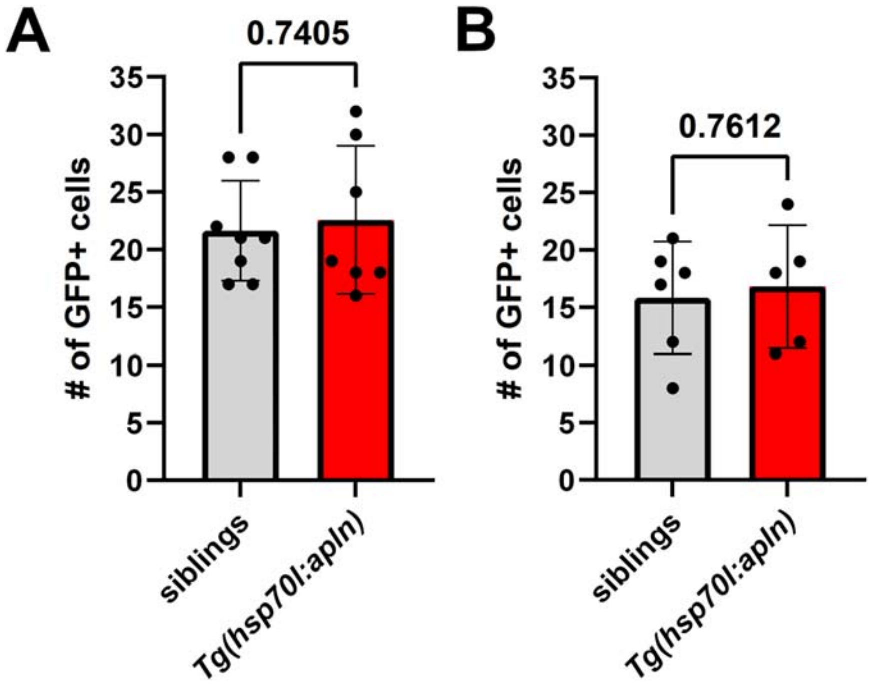

Figure EV5. Overexpression of *apln* does not influence HSPC specification and maintenance.

(**A**, **B**) Quantification of *itga2b:*GFP expressing HSPCs in the DA at 48 hpf upon heatshock induced global *apln* overexpression. (**A**) heatshock at 16 hpf, imaging at 48 hpf ($n = 15$, unpaired t test, $P = 0.7405$). (**B**) heatshock at 32 hpf, imaging at 48 hpf ($n = 11$, unpaired t test, $P = 0.7612$). HSPC hematopoietic stem and progenitor cell, DA dorsal aorta. Quantifications are displayed as mean ± SD.

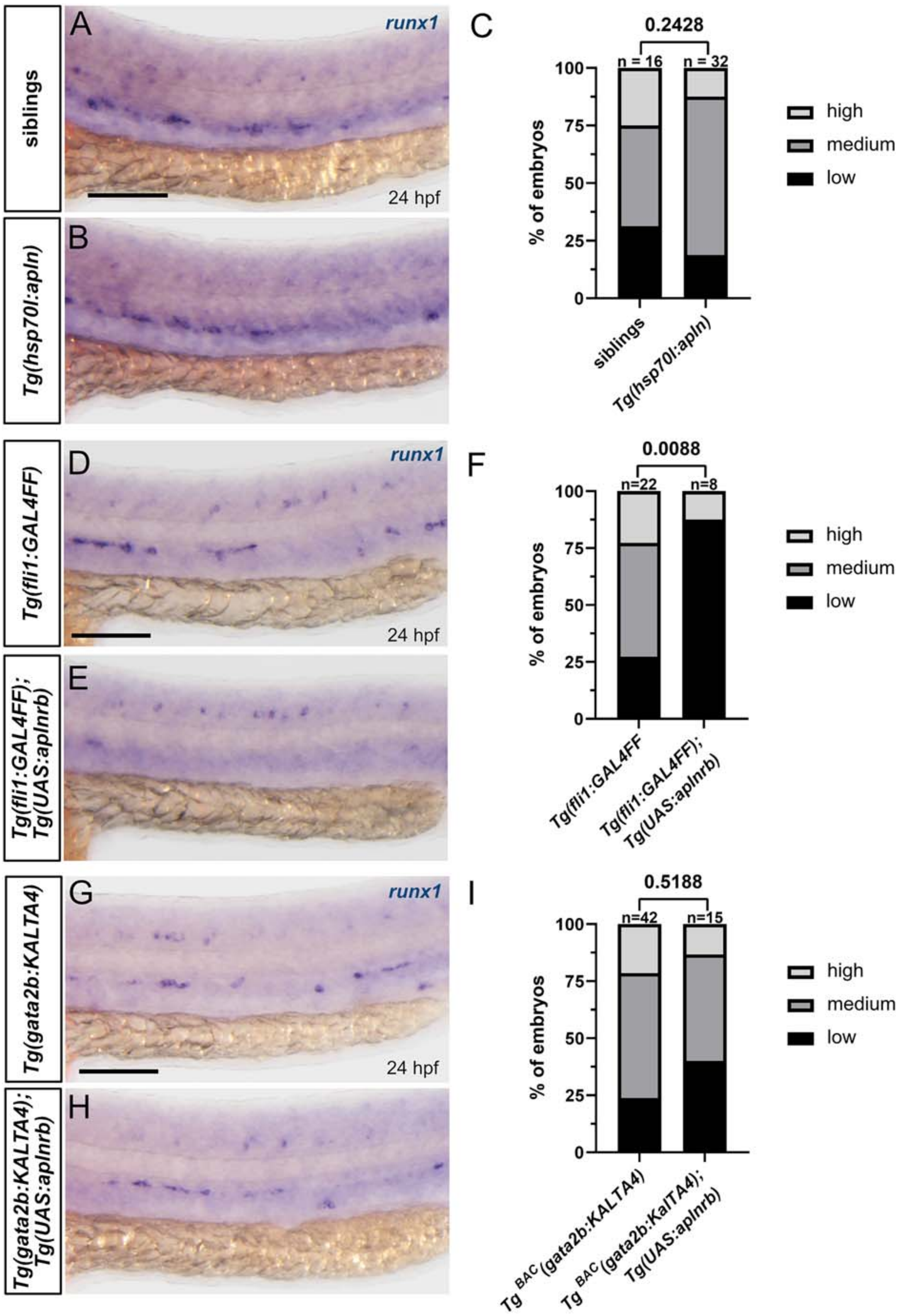
A
runx1
siblings
24 hpf
B
Tg(hsp70l:apln)
C
0.2428
n = 16
n = 32
100
75
50
25
0
% of embryos
high
medium
low
siblings
Tg(hsp70l:apln)
D
runx1
Tg(fli1:GAL4FF)
24 hpf
E
Tg(fli1:GAL4FF);
Tg(UAS:aplnrb)
F
0.0088
n=22
n=8
% of embryos
high
medium
low
Tg(fli1:GAL4FF
Tg(fli1:GAL4FF);
Tg(UAS:aplnrb)
G
runx1
Tg(gata2b:KALTA4)
24 hpf
H
Tg(gata2b:KALTA4);
Tg(UAS:aplnrb)
I
0.5188
n=42
n=15
% of embryos
high
medium
low
Tg BAC (gata2b:KALTA4)
Tg BAC (gata2b:KalTA4);
Tg(UAS:aplnrb)

© The Author(s)

Figure EV6. Forced expression of *aplnrb* in the vasculature disturbs HE specification.

(**A**, **B**, **D**, **E**, **G**, **H**) WISH for *runx1* at 24 hpf upon *apln/aplnrb* overexpression. Images of the trunk region above the yolk extension. (**A**, **B**) WISH for *runx1* in siblings or *hsp70l:apln* (heatshock inducible global overexpression of *apln*) zebrafish embryos. The heatshock was induced at 16 hpf. Embryos with global *apln* overexpression display no differences in *runx1* expression compared with control siblings. (**C**) Phenotypic distribution plot of embryos in (**A**, **B**) scored with low, medium and high *runx1* expression ($n = 48$, Chi-square test, $P = 0.2428$). (**D**, **E**) WISH for *runx1* in *fli1:*Gal4FF (control) or *fli1:*Galf4FF/*UAS:*aplnrb (pan-vascular *aplnrb* overexpression) zebrafish embryos. Embryos with vascular *aplnrb* overexpression display a drastic reduction in *runx1* expression compared with control siblings. (**F**) Phenotypic distribution plot of embryos in (**D**, **E**) scored with low, medium and high *runx1* expression ($n = 30$, Chi-square test, $P = 0.0088$). (**G**, **H**) WISH for *runx1* in *gata2b:*KaltA4 (control) or *gata2b:*KaltA4/*UAS:*aplnrb (hemogenic endothelium specific overexpression of *aplnrb*) zebrafish embryos. Embryos with HE specific *aplnrb* overexpression display no differences in *runx1* expression compared with control siblings. (**I**) Phenotypic distribution plot of embryos in (**G**, **H**) scored with low, medium and high *runx1* expression ($n = 57$, Chi-square test, $P = 0.5188$). HE hemogenic endothelium. Quantifications are displayed as mean ± SD. Scale bars: 100 µm.

© The Author(s)

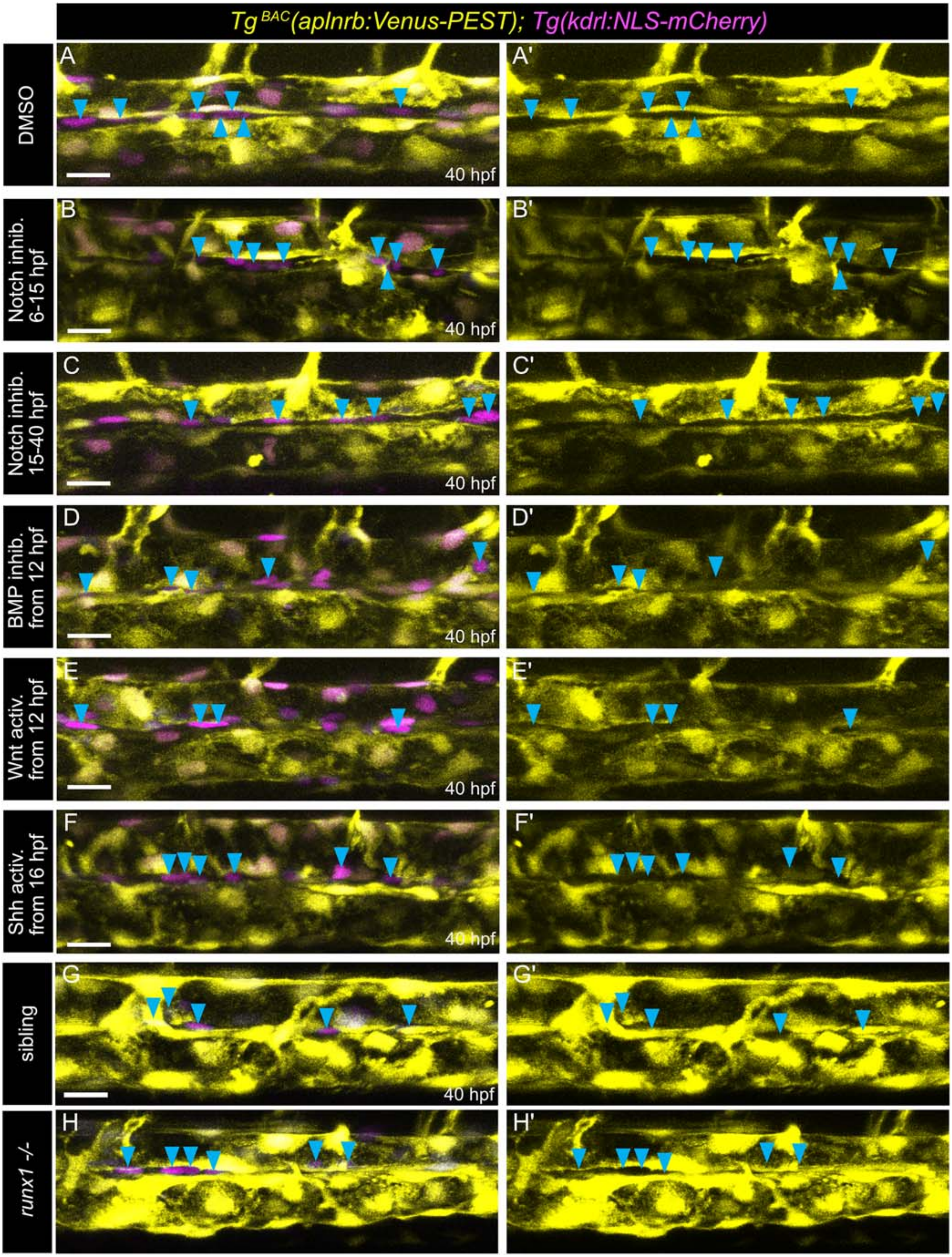
Tg BAC(aplnrb:Venus-PEST); Tg(kdrl:NLS-mCherry)
DMSO
Notch inhib. 6-15 hpf
Notch inhib. 15-40 hpf
BMP inhib. from 12 hpf
Wnt activ. from 12 hpf
Shh activ. from 16 hpf
sibling
runx1 -/-
A
A'
B
B'
C
C'
D
D'
E
E'
F
F'
G
G'
H
H'
40 hpf

 © The Author(s)

Figure EV7. *aplnrb* repression in HEC is independent of major regulators of embryonic hematopoiesis.

(**A–H′**) Confocal projection images of the trunk region of *Tg^BAC^(aplnrb:Venus-PEST); Tg(kdrl:NLS-mCherry)* double transgenic zebrafish embryos. *aplnrb:*Venus-PEST negative HECs can be detected in the ventral DA (cyan arrowheads). (**A**, **A′**) Trunk region of DMSO treated control embryos corresponding to (**B–F′**), imaging at 40 hpf. (**B**, **B′**) Trunk region of Notch inhibitor (1 μM RO4929097) treated embryos, treatment from 6–15 hpf, imaging at 40 hpf. WT-like *aplnrb*:Venus-PEST expression in the ventral DA can be observed. (**C**, **C′**) Trunk region of Notch inhibitor (1 μM RO4929097) treated embryos, treatment from 15–40 hpf, imaging at 40 hpf. WT-like *aplnrb*:Venus-PEST expression in the ventral DA can be observed. (**D**, **D′**) Trunk region of BMP inhibitor (10 μM DMH1) treated embryos, treatment from 12–40 hpf, imaging at 40 hpf. WT-like *aplnrb*:Venus-PEST expression in the ventral DA can be observed. (**E**, **E′**) Trunk region of Wnt activator (0.15 M LiCl) treated embryos, treatment from 12–40 hpf, imaging at 40 hpf. WT-like *aplnrb*:Venus-PEST expression in the ventral DA can be observed. (**F–F′**) Trunk region of Shh activator (20 μM Purmorphamine) treated embryos, treatment from 16–40 hpf, imaging at 40 hpf. WT-like *aplnrb*:Venus-PEST expression in the ventral DA can be observed. (**G–H′**) Trunk region of siblings and *runx1 −/−* mutant zebrafish embryos at 40 hpf. WT-like *aplnrb*:Venus-PEST expression can be observed in the ventral DA of *runx1 −/−* mutant embryos. HE hemogenic endothelium, EC endothelial cell, DA dorsal aorta. Scale bars: 20 μm.

© The Author(s)

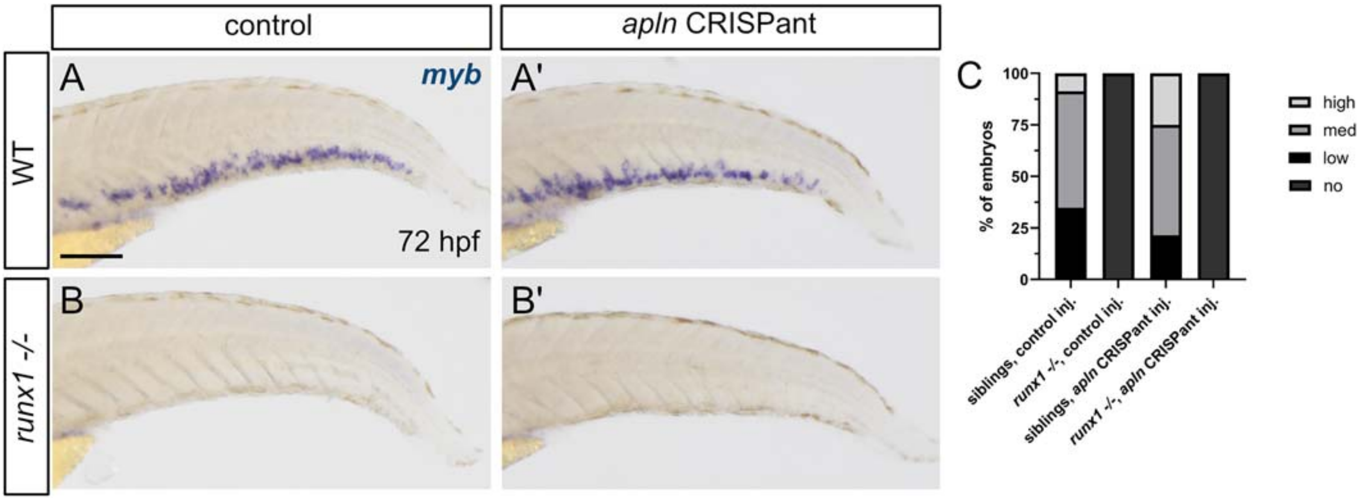

Figure EV8. Elevated HSPC numbers upon loss of Apelin signaling are dependent on Runx1 function.

(**A**, **B**) WISH for *myb* at 72 hpf in *runx1 -/-* mutant larvae and siblings. Images of the CHT region. (**A**, **A'**) WISH for *myb* in control injected and *apln* CRISPR injected wild-type siblings. Loss of Apelin signaling results in increased *myb* expression in the CHT compared with control injected siblings. (**B**, **B'**) WISH for *myb* in control injected and *apln* CRISPR injected *runx1 -/-* mutant larvae. Loss of Apelin signaling does not rescue *myb* expression in the CHT. (**C**) Phenotypic distribution plot of embryos in (**A**, **B**) scored for no, low, medium and high *myb* expression ($n = 71$). CHT caudal hematopoietic tissue. Scale bars: 200 µm.

 © The Author(s)
